# Endovascular Treatment of Hemoptysis: Does Bronchial Artery Embolization Fit All?

**DOI:** 10.3390/diagnostics16172844

**Published:** 2026-09-04

**Authors:** Francesco Rocco Bertuccio, Lucrezia Pisanu, Giulia Accordino, Matteo Bosio, Pietro Quaretti, Nicola Cionfoli, Riccardo Corti, Mauro Antonio D’Agostino, Giulia Maria Stella, Angelo Guido Corsico, Alessandro Cascina

**Affiliations:** 1 Department of Internal Medicine and Medical Therapeutics, University of Pavia Medical School, 27100 Pavia, Italy; 2 Unit of Respiratory Diseases, Cardiothoracic and Vascular Department, Fondazione IRCCS Policlinico San Matteo, 27100 Pavia, Italy; 3 Interventional Radiology Unit, Fondazione IRCCS Policlinico San Matteo, 27100 Pavia, Italy

**Keywords:** hemoptysis, tracheal varices, superior vena cava obstruction

## Abstract

Hemoptysis is usually attributed to bleeding from bronchial or non-bronchial systemic arterial circulation. Venous airway bleeding is rarely considered, although it requires a different diagnostic and therapeutic approach. A 64-year-old man with a large retrosternal multinodular goiter was admitted for recurrent hemoptysis. Computed tomography showed left lower lobe consolidation and marked compression of the superior vena cava and brachiocephalic veins, with extensive mediastinal and chest-wall collateralization. Initial bronchoscopy suggested bleeding from the left lower lobe, and left bronchial artery embolization achieved only transient hemostasis. Recurrent bleeding within 24 h prompted repeat bronchoscopy, which demonstrated multiple submucosal tracheal varices; narrow-band imaging further delineated their vascular architecture. Integration of bronchoscopic and radiological findings established the diagnosis of venous hemoptysis secondary to benign central venous obstruction. Bilateral brachiocephalic vein stenting restored venous drainage and produced immediate and sustained resolution of hemoptysis. Subsequent thyroidectomy was performed without complications. Tracheal varices should be considered when hemoptysis recurs despite technically adequate bronchial artery embolization, particularly in the presence of central venous obstruction and collateral circulation. Definitive treatment should target the underlying venous hypertension by restoring central venous outflow.

**Figure 1:**
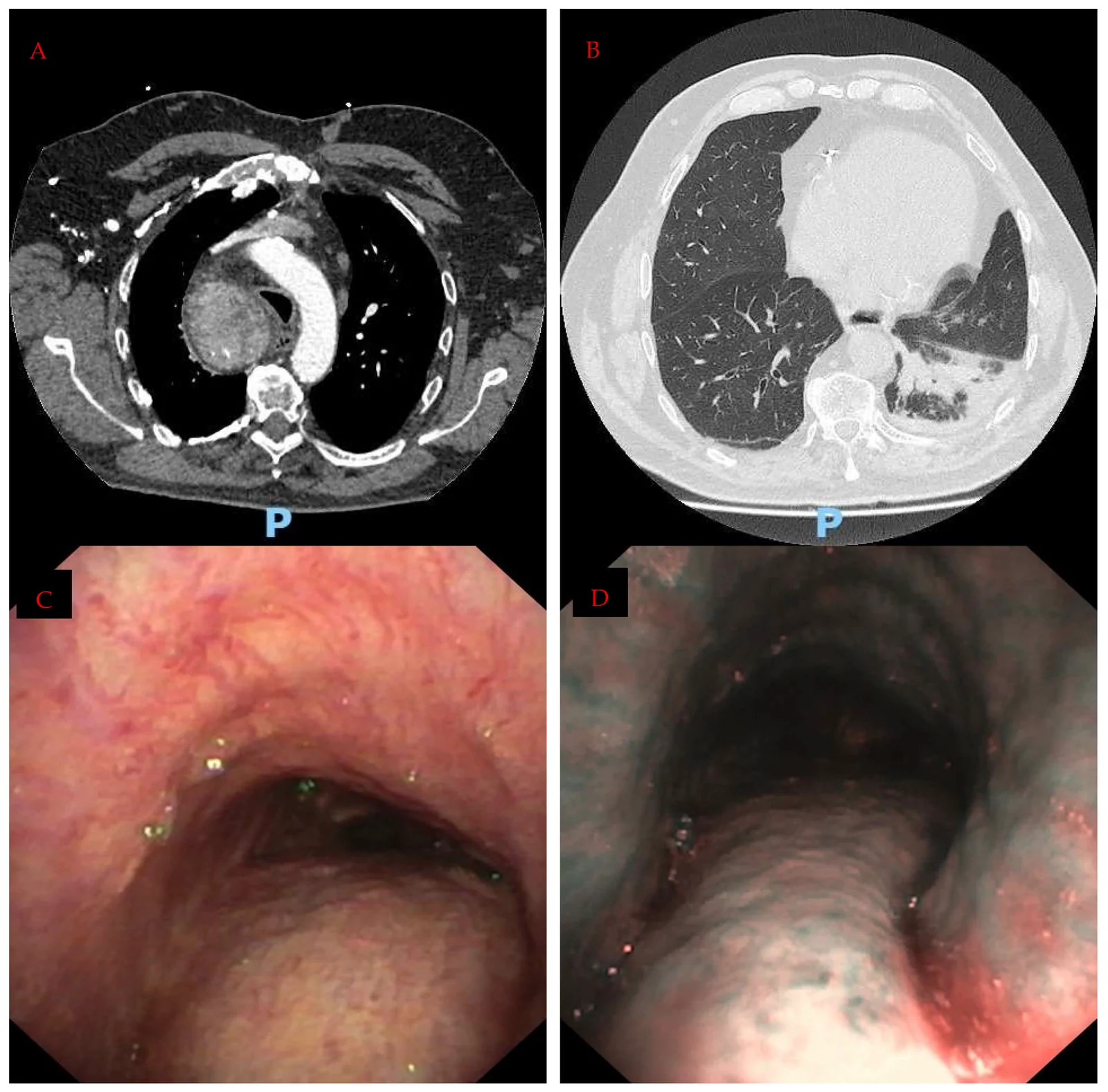
Hemoptysis most commonly originates from the bronchial or non-bronchial systemic arterial circulation, and current diagnostic and interventional strategies are therefore largely directed toward identifying and controlling arterial sources of bleeding. Venous hemoptysis is far less frequently recognized, although its distinction from arterial bleeding is clinically relevant because the underlying hemodynamic abnormality cannot be corrected by arterial embolization. Central venous obstruction may produce marked collateralization and increased pressure within the mediastinal, thyroidal and tracheoesophageal venous plexuses. Progressive dilation of these networks can result in fragile submucosal tracheal varices, which may rupture into the airway and present as recurrent hemoptysis. Their diagnosis can be challenging, particularly when concurrent pulmonary abnormalities suggest a more conventional peripheral bleeding source. We describe a case of tracheal varices caused by benign superior vena cava and brachiocephalic venous obstruction from a large retrosternal goiter, successfully treated by restoration of central venous drainage. A 64-year-old man was admitted for recurrent moderate-volume hemoptysis. During the preceding days, he had developed productive cough and mild dyspnea without fever or chest pain. On the day of admission, three further episodes of hemoptysis prompted presentation to the Emergency Department. He was a former smoker with a 21.5 pack-year history and previous occupational exposure to construction dust. His medical history was notable for a multinodular goiter with retrosternal extension to the posterolateral margin of the aortic arch. Total thyroidectomy had previously been recommended but declined; subsequent multidisciplinary reassessment considered elective surgery to carry prohibitive risk. Additional comorbidities included ischemic heart disease treated with coronary artery bypass grafting, hypertension, peripheral arterial disease, type 2 diabetes mellitus, and obstructive sleep apnea. On admission, the patient was hemodynamically stable, afebrile, and mildly dyspneic, with an oxygen saturation of 94% on room air. Neck swelling was evident. Hemoglobin and platelet count were normal, inflammatory biomarkers were not elevated, and renal and hepatic function were preserved. An extensive autoimmune panel was negative. Chest radiography showed widening of the upper mediastinum, consistent with the known retrosternal goiter. Transthoracic echocardiography demonstrated preserved biventricular function without evidence of pulmonary hypertension. Contrast-enhanced computed tomography angiography excluded pulmonary embolism and acute aortic disease and showed no active arterial extravasation. Lung-window images demonstrated left lower lobe consolidation with air bronchograms (**B**), whereas mediastinal-window images revealed a large intrathoracic goiter with patchy vascularization and calcifications, causing severe compression of the superior vena cava and both brachiocephalic veins, with extensive mediastinal and chest-wall venous collateralization (**A**). Flexible bronchoscopy demonstrated blood clots throughout the tracheobronchial tree, predominantly within the left lower lobe bronchus, where a tenacious fibrin clot prevented complete distal inspection. No endobronchial lesion was identified. Because hemoptysis was ongoing and the bronchoscopic clot burden suggested a left lower lobe source, invasive angiographic evaluation was undertaken. Selective angiography of the bronchial and other systemic arterial territories showed no arterial hypertrophy, abnormal vascularity, systemic-to-pulmonary shunting, pseudoaneurysm, or contrast extravasation. The left bronchial artery was selectively catheterized using a JR4 guiding catheter and an Echelon microcatheter. Despite the absence of angiographic evidence of active bleeding, empirical embolization was performed using a 1:1 Glubran-Lipiodol mixture, based on the persistent hemoptysis and the bronchoscopic localization of the clot burden. Completion angiography confirmed exclusion of the treated vessel. Immediate hemostasis was achieved, but hemoptysis recurred within 24 h. Repeat bronchoscopy demonstrated multiple collapsed submucosal varices along the membranous trachea (Panel (**C**); bronchoscopic appearance of tracheal varices. White-light bronchoscopy showing collapsed submucosal varices on the membranous trachea). Narrow-band imaging enhanced their vascular architecture and revealed additional lesions extending along the cartilaginous trachea (Panel (**D**); narrow-band imaging view emphasizing the vascular pattern along the cartilaginous trachea). Bronchoalveolar lavage cultures, including bacterial, mycobacterial, and fungal studies, were negative. Integration of the bronchoscopic and radiological findings established the diagnosis of venous hemoptysis caused by tracheal varices secondary to benign superior vena cava and brachiocephalic venous obstruction from the retrosternal goiter.

**Figure 2:**
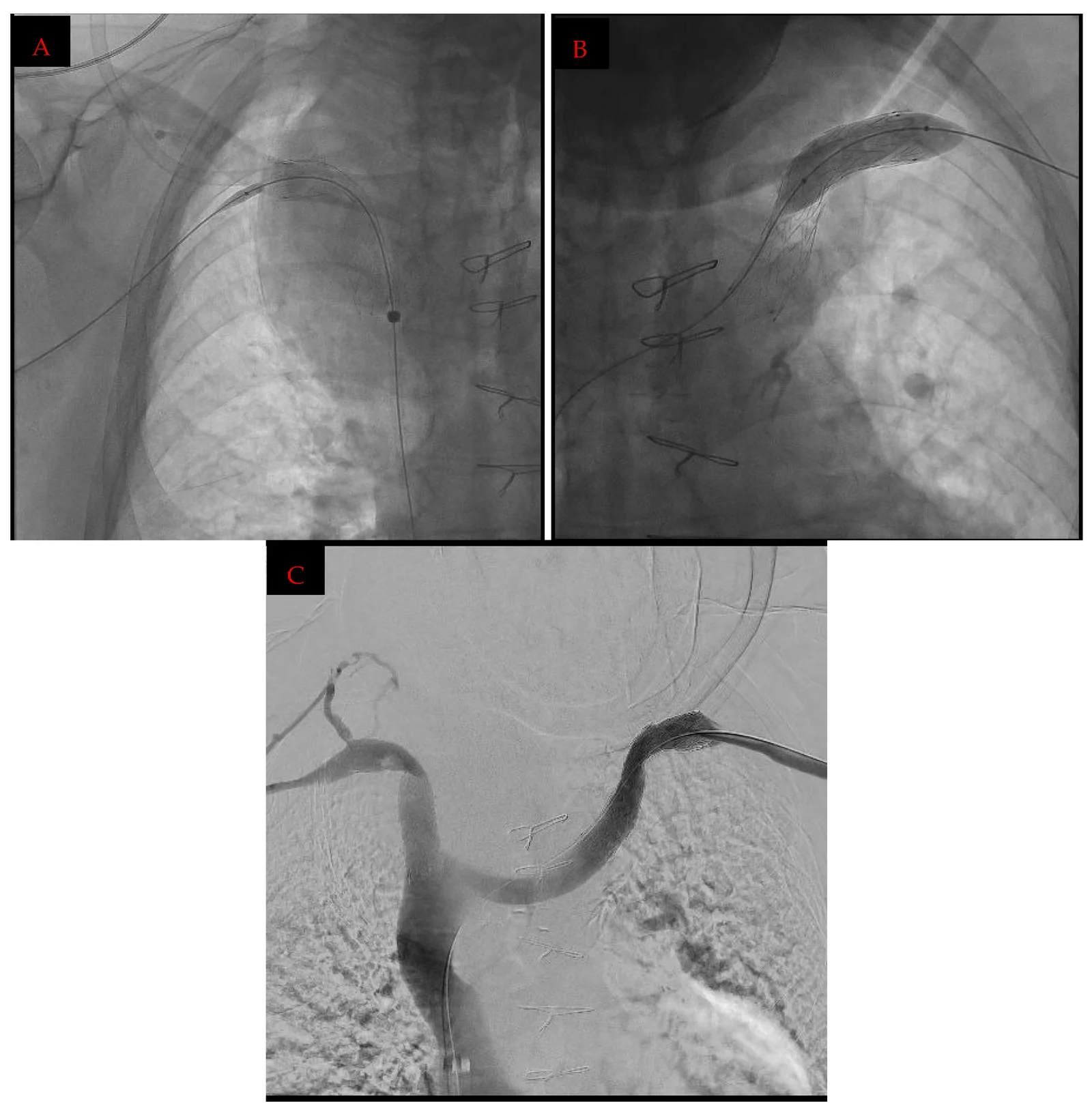
Following multidisciplinary discussion, endovascular recanalization and bilateral brachiocephalic vein stenting with self-expandable PTFE-covered stent grafts were performed. The procedure was performed through right common femoral and bilateral basilic venous accesses. Diagnostic cavography demonstrated bilateral occlusion of the subclavian-brachiocephalic venous segments with extensive thoracic and cervicothyroid collateral circulation, while the superior vena cava remained patent. Following recanalization, 8 mm balloon angioplasty resulted in immediate recoil. A 14 × 80 mm WRAPSODY PTFE-covered stent graft was therefore deployed on the right and a 16 × 80 mm WRAPSODY PTFE-covered stent graft on the left; both were initially dilated to 8 mm and subsequently post-dilated to 12 mm. Completion venography demonstrated bilateral patency, restored central venous drainage, and marked reduction in the collateral circulation. No venous rupture or contrast extravasation was observed. Hemoptysis resolved immediately after the procedure. (Panel (**A**): upper extremity spot X-ray during stentgraft deployment on the right anonymous vein. Panel (**B**): balloon dilation inside the stentgraft on the left anonymous vein; (**C**): bilateral patency of subclavian-brachiocephalic venous segment after stent placement at venography). Hemoptysis resolved immediately. Subsequent upper gastrointestinal endoscopy demonstrated non-bleeding F1 downhill esophageal varices, supporting the presence of chronic central venous hypertension. During the initial intraprocedural venographic assessment, a concomitant left internal jugular vein thrombosis was identified before stent deployment and was therefore considered pre-existing rather than procedure-related. The patient was subsequently discharged on low-molecular-weight heparin at a dose of 4000 IU (enoxaparin) subcutaneously once daily. Given the recent recurrent hemoptysis, prophylactic-dose rather than therapeutic-dose anticoagulation was initially selected after an individualized assessment of thrombotic and bleeding risks. In our patient, angioplasty alone resulted in immediate recoil because of the chronic extrinsic compression. PTFE-covered stent grafts were therefore selected to provide greater luminal stability and optimize primary patency, consistent with previous evidence comparing stent grafts with angioplasty and bare-metal stents in central venous obstruction [1]. Their use was not prompted by suspected venous rupture. Follow-up computed tomography angiography confirmed persistent stent patency, and no further hemoptysis occurred. Following restoration of central venous drainage, the anticipated operative risk was reassessed. Decompression of the cervicomediastinal venous collateral network was considered to reduce the risks of perioperative bleeding, airway compromise, and anesthetic complications. After repeat multidisciplinary evaluation, total thyroidectomy was considered feasible and was subsequently performed without anesthetic or airway-related complications. Venous hemoptysis represents a distinct and frequently overlooked mechanism of airway bleeding. Although most clinically significant episodes arise from the bronchial or non-bronchial systemic arterial circulation, central venous obstruction may generate extensive collateral networks involving the mediastinal, thyroidal, tracheal, and esophageal venous plexuses. Superior vena cava obstruction is most commonly associated with malignancy, but benign causes such as central venous catheters, thrombosis, mediastinal fibrosis, and compressive retrosternal goiters are increasingly recognized [2,3,4,5]. The anatomical basis of tracheal varices is particularly relevant. The venous plexuses of the proximal trachea and upper esophagus communicate with the inferior thyroid venous system, which normally drains into the brachiocephalic veins. Obstruction of this pathway increases pressure within the thyroidal and paratracheal venous networks and redirects blood through collateral vessels. Progressive dilation of these vessels may result in fragile submucosal tracheal varices that can rupture into the airway. The associated presence of proximal, or “downhill” esophageal varices reflects the same underlying hemodynamic mechanism [5,6,7,8,9]. The airways have a dual and highly interconnected venous drainage. The extrapulmonary bronchial veins drain the main bronchi and hilar structures into the azygos system on the right and into the hemiazygos, accessory hemiazygos, or left superior intercostal veins on the left. By contrast, much of the intrapulmonary bronchial circulation drains through deep bronchial veins into the pulmonary veins and ultimately the left atrium. Proximally, the tracheal and upper esophageal venous plexuses communicate with the inferior thyroid veins and brachiocephalic veins. This anatomy permits collateral flow between the systemic and pulmonary venous compartments; when outflow is obstructed or venous pressure rises, flow may be redirected through enlarged submucosal channels, producing tracheobronchial varices [10,11]. Portal hypertension may also, although rarely, produce tracheobronchial varices through portosystemic collateral pathways [12]. Pulmonary venous hypertension denotes abnormally elevated pressure within the pulmonary venous and left atrial compartment. It most commonly results from left-sided cardiac disease, including mitral valve disease, left ventricular systolic or diastolic dysfunction, or left atrial obstruction. Focal pulmonary venous hypertension may instead result from congenital or acquired pulmonary vein stenosis or occlusion, including after atrial fibrillation ablation, lung surgery or transplantation, or fibrosing mediastinitis. When elevated left atrial pressure produces pulmonary hypertension, the current hemodynamic definition of post-capillary pulmonary hypertension is a mean pulmonary arterial pressure >20 mmHg with a pulmonary arterial wedge pressure >15 mmHg. Back-pressure transmitted through bronchopulmonary venous anastomoses may cause submucosal bronchial venous engorgement, pulmonary edema, alveolar hemorrhage, bronchial varices, and hemoptysis [13,14,15]. This mechanism should not be confused with that of the present case: normal echocardiographic estimates of pulmonary arterial pressure did not exclude systemic central venous hypertension upstream of the superior vena cava and brachiocephalic vein obstruction. Chronic lung diseases should be distinguished from true venous hypertension because their vascular remodeling is predominantly arterial. In bronchiectasis and chronic bronchitis, recurrent infection and inflammation promote angiogenic remodeling, hypertrophy and tortuosity of the bronchial and non-bronchial systemic arteries, and the development of fragile systemic-to-pulmonary anastomoses. Similar collateral recruitment may accompany architectural distortion and chronic hypoxemia in fibrotic lung disease. These vessels are exposed to systemic pressure and may rupture into the airway. Thus, hemoptysis in these conditions is generally arterial rather than venous. In pulmonary fibrosis, hemoptysis is not typical of uncomplicated disease and should also prompt evaluation for traction bronchiectasis, superimposed infection or mycetoma, malignancy, thromboembolic disease, or diffuse alveolar hemorrhage [16,17,18]. The principal conditions associated with tracheobronchial vascular enlargement and hemoptysis are summarized in Table A1. Recognition of this venous origin has immediate diagnostic and therapeutic implications. Bronchial artery embolization is highly effective for arterial hemoptysis but cannot correct bleeding caused by impaired venous outflow. In this setting, early recurrence after technically adequate embolization should not automatically be interpreted as procedural failure or lead to repeated embolization. Instead, it should prompt reassessment of the presumed vascular mechanism. Contrast-enhanced computed tomography is essential for identifying central venous obstruction and collateral circulation, while bronchoscopy allows direct visualization of submucosal vascular lesions. Narrow-band imaging may further support diagnosis by enhancing the superficial vascular pattern of tracheal varices. In the present case, however, the tracheal varices appeared collapsed and were not directly observed during active bleeding. Although the overall clinical, radiological, and bronchoscopic findings strongly supported a venous origin, this diagnosis remained inferential; therefore, a concomitant occult arterial source could not be excluded with absolute certainty. Once venous hemoptysis has been identified, treatment should target the underlying venous hypertension. Endovascular recanalization and stenting can restore physiological drainage, decompress collateral networks, and achieve durable hemostasis [1,3,4]. In benign compressive disease, venous decompression may also reduce cervicomediastinal congestion and facilitate subsequent definitive surgery. In the present case, bilateral brachiocephalic vein stenting resulted in immediate and sustained cessation of hemoptysis and subsequently allowed uncomplicated thyroidectomy. Although tracheal varices associated with superior vena cava obstruction or large goiters have previously been reported [6,7,8,9,19], this case illustrates the successful use of bilateral brachiocephalic vein stenting as a mechanism-directed treatment for recurrent venous hemoptysis. Persistent or recurrent bleeding after adequate bronchial artery embolization should therefore prompt reconsideration of the assumption that all hemoptysis is arterial. Identifying central venous obstruction may fundamentally change both diagnosis and treatment.

## Data Availability

All data relevant to this case study are included in the article. Additional information may be obtained from the corresponding author upon reasonable request, subject to patient confidentiality and applicable data protection regulations.

## Appendix A

**Table A1:** Selected causes of tracheobronchial vascular enlargement, congestion, and hemoptysis.

Pathophysiologic Group	Representative Causes	Mechanism and Characteristic Vascular Manifestations
Central systemic venous hypertension	Superior vena cava or brachiocephalic vein obstruction from malignancy, catheter- or device-related stenosis/thrombosis, fibrosing mediastinitis, retrosternal goiter, or another mediastinal mass	Increased pressure within the azygos, internal thoracic, thyroidal, mediastinal, and tracheoesophageal venous plexuses, producing chest-wall collateral vessels, tracheobronchial varices, and downhill esophageal varices
Portal hypertension	Cirrhosis, portal vein thrombosis, or extrahepatic portal venous obstruction	Portosystemic collateral flow through esophageal, paraesophageal, and azygos pathways may rarely extend to the bronchial or tracheal venous plexuses, producing varices and hemoptysis
Pulmonary venous hypertension due to cardiac disease	Mitral stenosis or regurgitation, left ventricular systolic or diastolic dysfunction, and left atrial obstruction	Elevated left atrial and pulmonary venous pressure is transmitted through bronchopulmonary venous anastomoses, causing bronchial venous engorgement, pulmonary edema, alveolar hemorrhage, or hemoptysis
Focal pulmonary venous obstruction	Congenital pulmonary vein stenosis or atresia; stenosis following atrial fibrillation ablation, lung surgery, or transplantation; fibrosing mediastinitis or tumor	Regional venous hypertension with bronchial-pulmonary venous collateralization, unilateral edema, pulmonary infarction, alveolar hemorrhage, or bronchial varices
Chronic airway or parenchymal disease, predominantly arterial	Bronchiectasis, cystic fibrosis, chronic bronchitis/COPD, tuberculosis or NTM disease, aspergillosis, and pulmonary fibrosis	Chronic inflammation, hypoxia, and architectural distortion produce hypertrophied bronchial and non-bronchial systemic arteries, neovascularization, and fragile systemic-to-pulmonary shunts
Pulmonary arterial or congenital vascular disease, predominantly arterial	Chronic thromboembolic pulmonary hypertension, severe pulmonary arterial hypertension, pulmonary artery interruption or atresia, and cyanotic congenital heart disease	Reduced pulmonary perfusion or increased pulmonary vascular resistance recruits systemic bronchial collaterals, which may rupture under systemic pressure
